# Pembrolizumab-Induced Acute Skin Reaction: A Case Report and Review of Literature

**DOI:** 10.7759/cureus.26143

**Published:** 2022-06-21

**Authors:** Mathew Thomas, Ali Wazir, Aarati Poudel

**Affiliations:** 1 Internal Medicine, State University of New York Upstate Medical University, Syracuse, USA; 2 Oncology, State University of New York Upstate Medical University, Syracuse, USA; 3 Hematology and Oncology, Syracuse Veterans Affairs Medical Center, Syracuse, USA

**Keywords:** pd-l1, skin reaction, lung cancer, pembrolizumab, immune-checkpoint inhibitors

## Abstract

The development of immune checkpoint inhibitors is considered to be one of the most important advances in cancer treatment. Pembrolizumab is an immune checkpoint inhibitor against programmed death-ligand 1 (PD-L1) receptor that has demonstrated antineoplastic activity against various malignancies including non-small cell lung cancer, melanoma, and triple-negative breast cancer. Pembrolizumab has been associated with significant dermatological adverse reactions, referred to as immune-related adverse events. The cutaneous adverse effects can affect the quality of life of the patient and can result in dose reduction or even discontinuation of the treatment. Hence it is of utmost importance to have a comprehensive understanding of the cutaneous toxicities for prompt initiation of treatment.

We present the case of a 49-year-old male with metastatic non-small cell lung cancer (NSCLC) with 100% PD-L1 expression, who suffered a severe cutaneous reaction involving more than 95% of body surface area, following the first dose of pembrolizumab. He was treated with low-dose systemic steroids (prednisone 10 mg), to which he responded well. Since the patient showed excellent symptomatic and clinical response to pembrolizumab, it was not discontinued. The patient has not developed a rash with subsequent doses of pembrolizumab, and the steroids were tapered off.

## Introduction

Pembrolizumab is a United States Food and Drug Administration-approved drug used in the treatment of both solid tumor and lymphoid malignancies [1]. Pembrolizumab is an immune checkpoint inhibitor that inhibits the interaction between programmed death (PD-1) with its ligands PD-L1 and PD-L2, thereby leading to cytotoxic T cell-mediated cancer cell recognition and anti-tumor activity [1]. Regarding adverse reactions, it can affect any organ system, with the skin being one of the most commonly affected organs [2]. The cutaneous side effects can range from mild maculopapular rash to life-threatening Stevens-Johnson syndrome (SJS) and toxic epidermal necrolysis (TEN) [2].

## Case presentation

**Figure 1:**
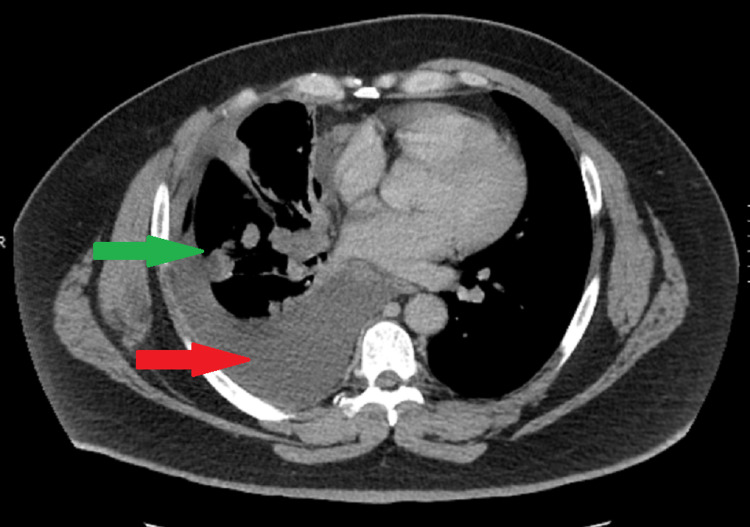
CT scan showing right-sided lung mass (green arrow) and pleural effusion (red arrow).

A 49-year-old male with no significant past medical history except for 14-year smoking history was evaluated for three months of shortness of breath. Imaging modalities including CT Chest and positron emission tomography (PET) scan revealed a right mediastinal lung mass of 3.4 x 4.1 cm, right-sided pleural effusion, and multiple mediastinal and hilar lymphadenopathy (Figure 1). Pathology of the lung mass biopsy showed poorly differentiated non-small cell lung cancer (NSCLC). Pleural fluid cytology was also positive for poorly differentiated NSCLC. The molecular markers tested positive for 100% PD-L1, but negative for epidermal growth factor receptor (EGFR), anaplastic lymphoma kinase (ALK), BRAF, and receptor tyrosine kinase (ROS-1).

**Figure 2:**
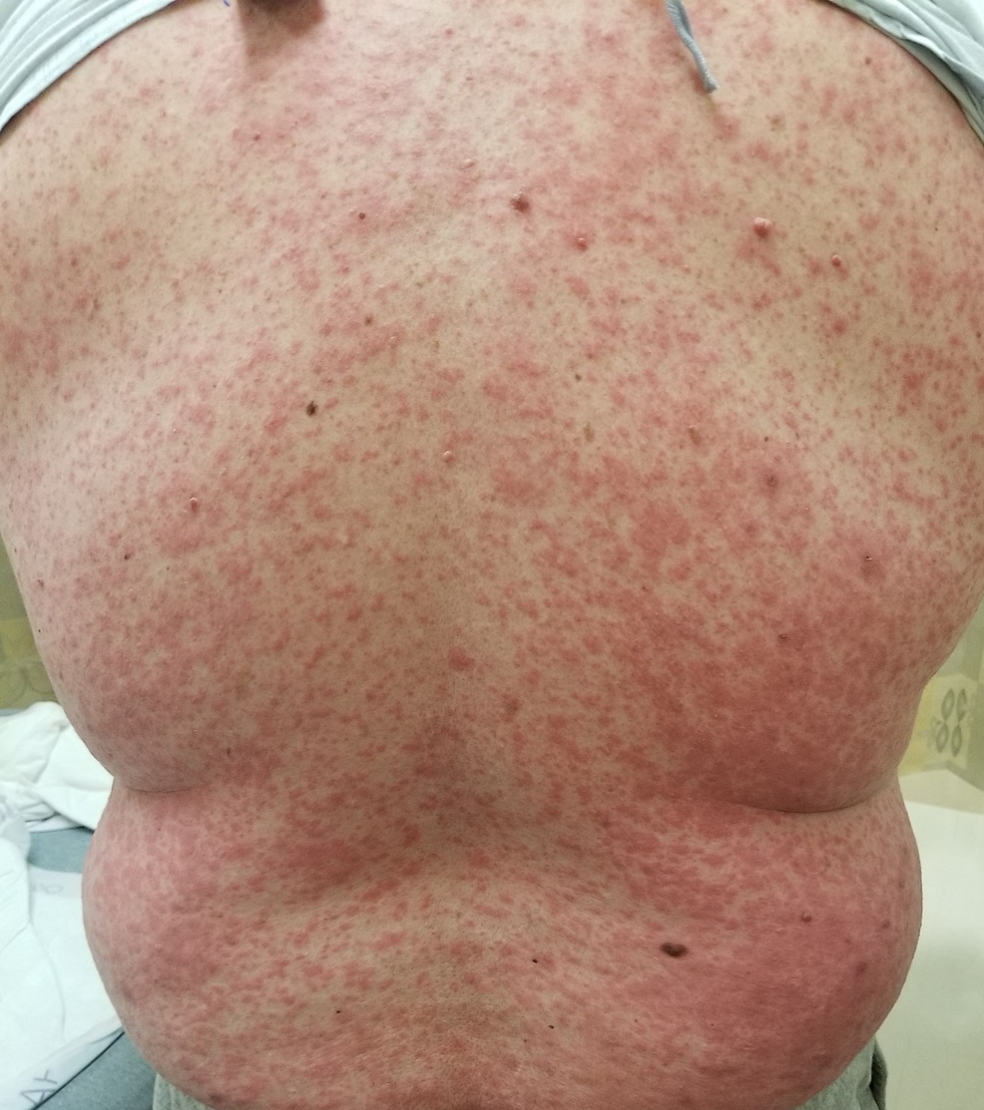
Diffuse maculopapular rash involving the trunk and back.

**Figure 3:**
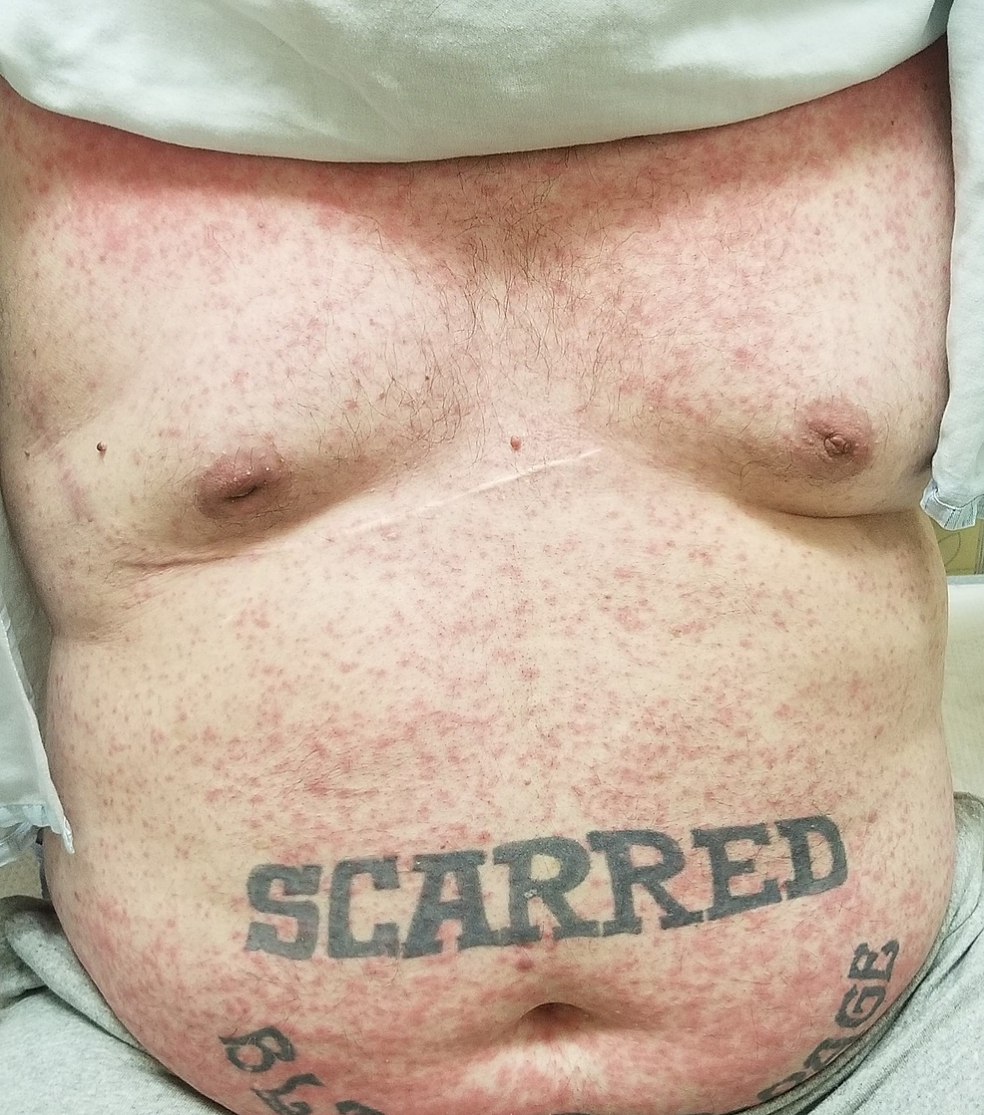
Diffuse maculopapular rash involving the chest and abdomen.

**Figure 4:**
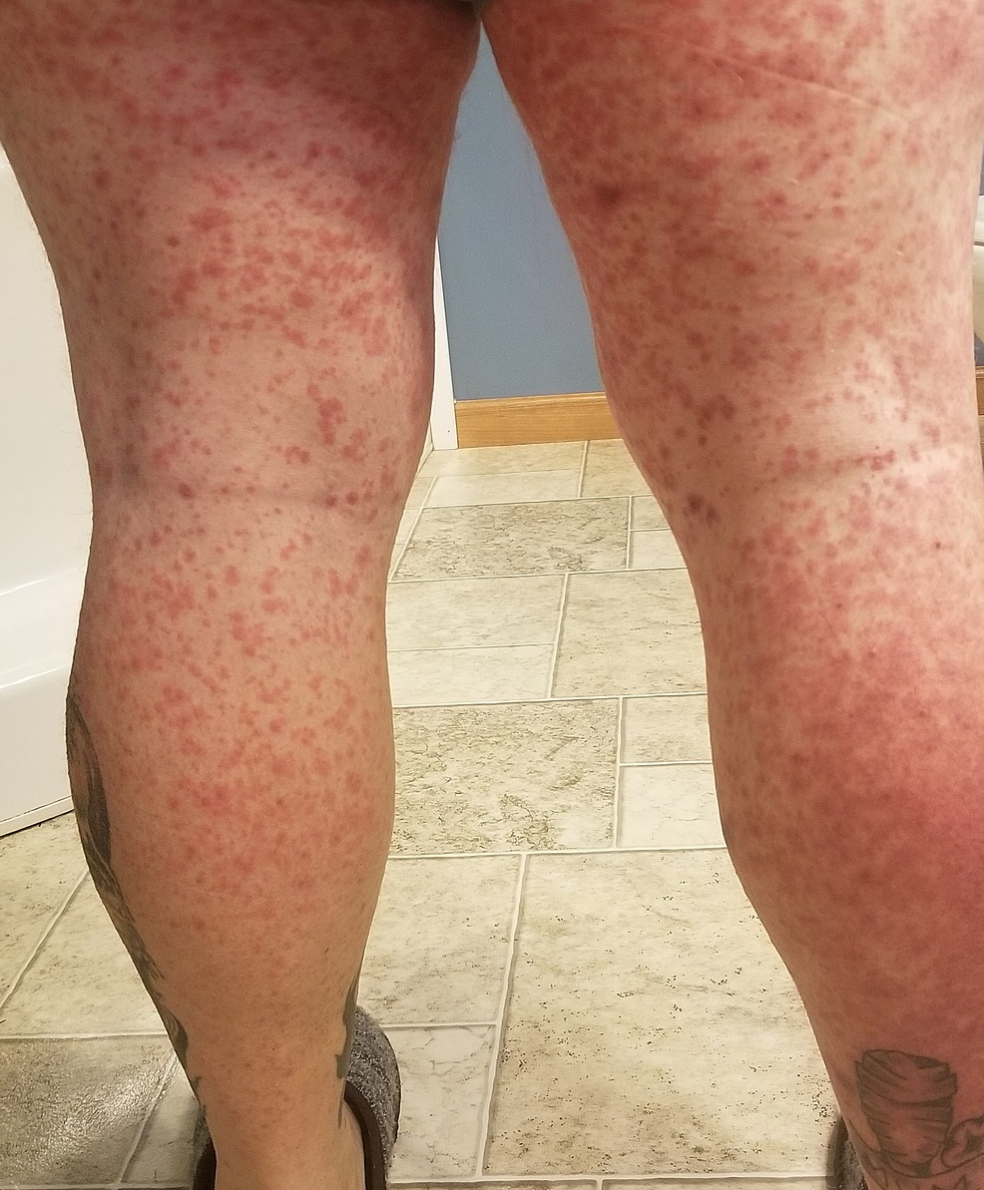
Diffuse maculopapular rash involving the posterior aspect of bilateral lower extremities.

**Figure 5:**
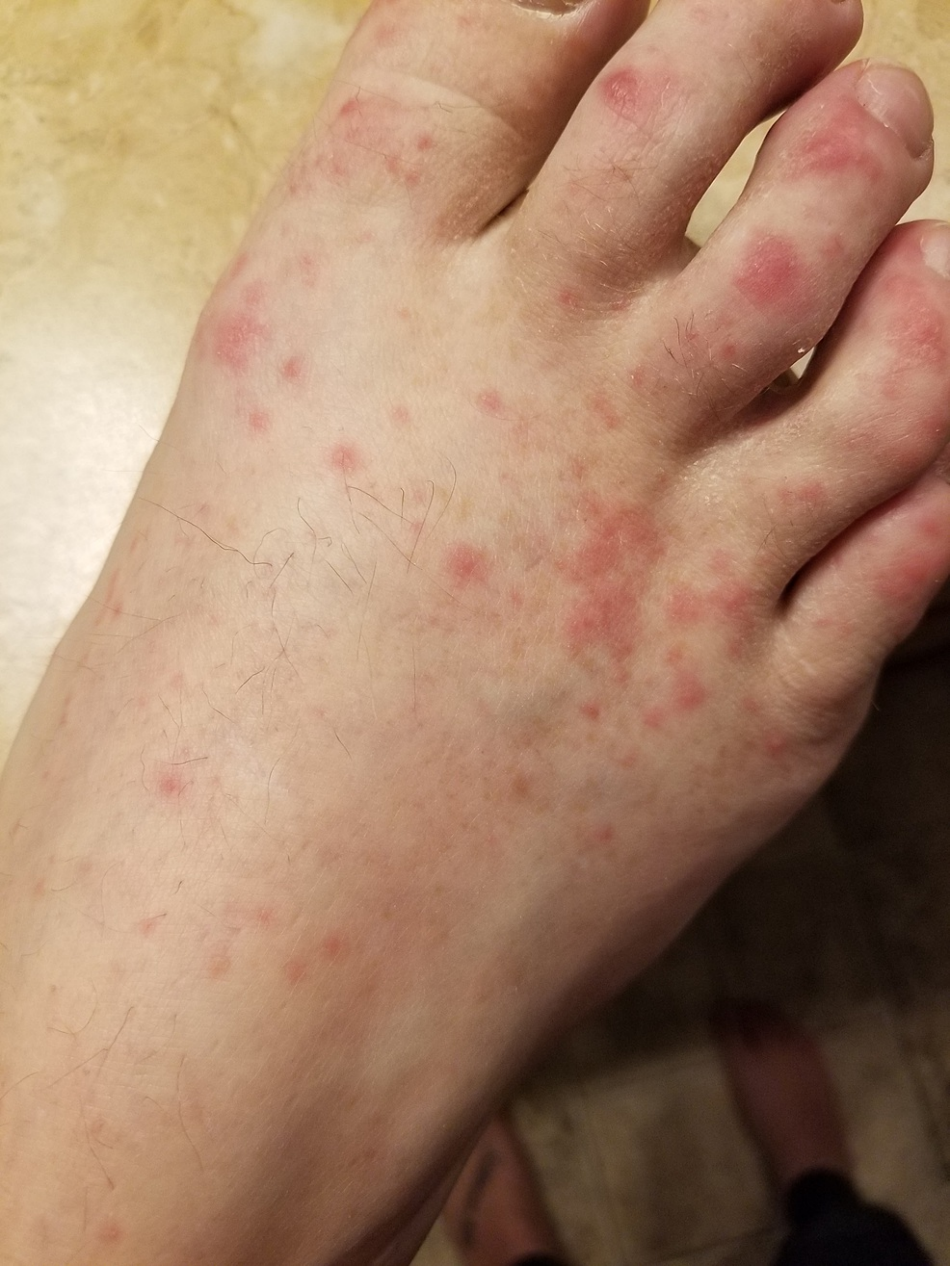
Maculopapular rash involving the right foot and toes.

**Figure 6:**
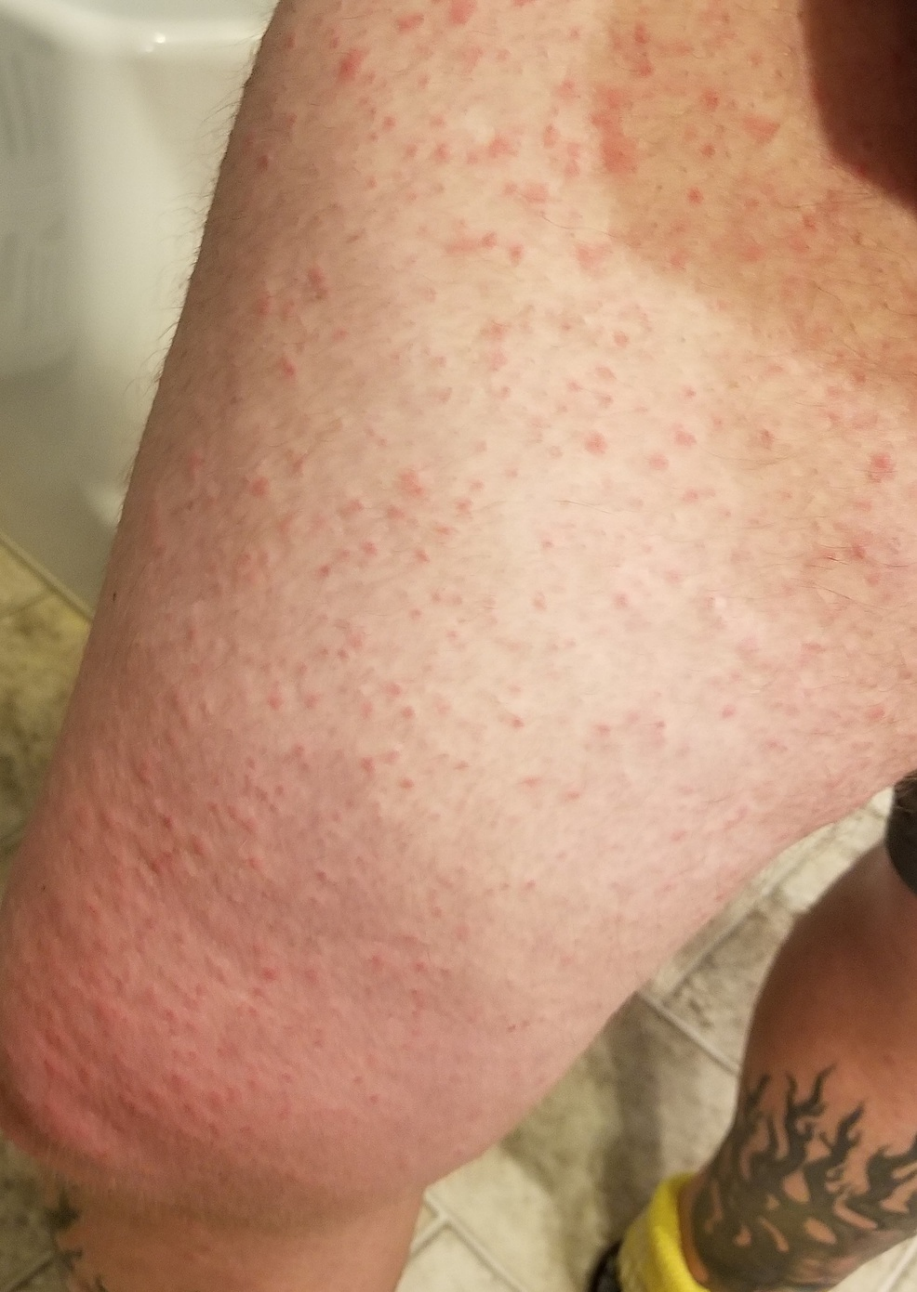
Diffuse maculopapular rash involving the anterolateral aspect of right thigh and knee.

His clinical course was complicated by recurrent pleural effusions, requiring multiple thoracenteses in two weeks. He was started on a combination of chemotherapy and immunotherapy with carboplatin, paclitaxel, and pembrolizumab (Keytruda®, Merck & Co., Inc., Rahway, USA). Unfortunately following the first cycle of the combination chemotherapy, he developed diffuse maculopapular skin rash involving >95% of the body surface area (Figures 2-6). Mucous membranes were not involved. The rash was not exfoliative in nature but was associated with pruritus. These rashes were considered to be an immune-related adverse reaction from the pembrolizumab. Hence, he was started on a low dose of 10 mg prednisone.

The patient showed excellent clinical response after the first cycle of chemotherapy-immunotherapy combination. His shortness of breath was dramatically improved and did not require any further thoracentesis. As the tumor had 100% expression of PD-L1, and the patient showed excellent clinical response, the decision was made to continue with the chemo-immunotherapy combination, along with 10 mg of prednisone for the prophylaxis of immune-related adverse reaction. Following the second cycle, he developed significant neuropathy in bilateral lower extremities, and hence carboplatin and paclitaxel were discontinued. However, he was continued on single-agent pembrolizumab, along with 10 mg of prednisone as mentioned above.

The rashes started to resolve with the steroids. Interestingly he did not develop any further rash after the fourth dose of pembrolizumab and eventually prednisone was tapered off. The patient continues to be on pembrolizumab maintenance therapy, without any steroids.

## Discussion

Lung cancer is the second most commonly diagnosed cancer after breast cancer and is the leading cause of cancer-related death in the world [3]. Non-small cell lung cancer accounts for more than 80-85% of lung cancer cases [4]. More than 60% of lung cancer patients present with locally advanced or metastatic disease at diagnosis [4]. Platinum-based chemotherapy remained the standard of care for metastatic non-small cell lung cancer (NSCLC) before the development of immune checkpoint inhibitors [1].

PD-L1 (programmed cell death ligand 1) is an immunoregulatory molecule that inhibits the cytotoxic CD8 T-cell activity, following the interaction with its PD-1 receptor [5]. Pembrolizumab is a humanized monoclonal IgG4 antibody that disrupts this interaction between PD-1 and PD-L1, thus leading to the recognition of cancer cells by the cytotoxic CD8 T-cells. In October 2016, FDA approved the use of pembrolizumab for previously untreated metastatic NSCLC with >50% PD-L1 expression, and for metastatic NSCLC with >1% PD-L1 expression who has progressed on platinum-based chemotherapy [1]. The addition of pembrolizumab to platinum-based chemotherapy in previously untreated metastatic NSCLC without EGFR or ALK mutations has shown to have longer overall survival and progression-free survival than chemotherapy alone [6].

Pembrolizumab is associated with a wide range of adverse events referred to as immune-related adverse events (ir- AES). These include hepatitis, colitis, pneumonitis, hypothyroidism, and cutaneous adverse events [7]. These adverse events are believed to be due to the lack of inhibition of T-cells [8]. The cutaneous adverse effects can affect the quality of life of the patients and can result in dose reduction or even discontinuation of the treatment [2,9]. The time of onset of the cutaneous toxicities can range from 4 to 10 months from the initiation of the treatment [10].

The overall cutaneous toxicities related to anti-PD1 antibodies are reported to be as high as 49% [11]. It can range from a mild rash to life-threatening toxic epidermal necrolysis (TEN) [12]. The three most commonly reported cutaneous adverse events related to ant-PD-L1 therapy include skin rash not otherwise specified, pruritus, and vitiligo [13, 14]. These cutaneous adverse events usually increase with increasing exposure to the treatment and thus require long-term monitoring [11]. A study by Sanlorenzo, M et al. investigated the development of cutaneous adverse events in a cohort of 83 patients treated with pembrolizumab [9]. In this study, maculopapular eruptions were the most common cutaneous reaction (occurring in up to 29%) and developed following the first dose of the pembrolizumab. None of these patients had mucous membrane involvement.

The cutaneous and mucocutaneous adverse events associated with PD-L1 inhibitors usually respond to topical and systemic steroids [9]. A single-institution study conducted by Coleman, E et al. investigated the dermatological adverse events associated with immune checkpoint inhibitors and their response to treatment [15]. Ninety-eight patients were included in the study, out of which 25% developed lichenoid reactions, 18% developed maculopapular or exanthematous reactions, and 17% developed psoriatic reactions. Seventy-five percent of the lesions had associated pruritus. Ninety percent of the rashes responded to topical steroids, while 20% required systemic steroids. A small percentage of the study population developed severe adverse reactions including immunobullous reactions, Stevens-Johnson syndrome, and erythroderma. They required aggressive treatment including acitretin, UVB (ultraviolet B) phototherapy, methotrexate, dapsone, and infliximab. Pruritus was managed with topical corticosteroids, topical camphor-menthol, antihistamines, gabapentin, pregabalin, and aprepitant. There was a temporary interruption of the treatment in 15.5% of the study population, and complete discontinuation in 8.7% of the population [15].

Another single-institution study of three cases of bullous lichen planus-like reaction following treatment with pembrolizumab was defined by Walkade DV [16]. Two patients had metastatic NSCLC and the third patient had metastatic melanoma. All of them had <95% of body surface area involved and resulted in interruption of treatment with pembrolizumab. There are other similar reported cases of adverse skin reactions including glossitis, bullous pemphigoid, and tumoral melanosis, treated with topical and systemic steroids, but resulted in disruption of treatment with pembrolizumab [17-20]. Our case is unique in the fact that he had >95% of body surface area involved, and pembrolizumab was never stopped.

## Conclusions

Pembrolizumab is an immune checkpoint inhibitor against PD-L1 and is widely used in the treatment of lung cancer. Pembrolizumab has been associated with immune-related cutaneous adverse events. Early identification of these cutaneous adverse events is of extreme importance. However, not every case will require high-dose steroids, and/or interruption of therapy. Our patient required only a short course of low-dose steroids without interruption of treatment with pembrolizumab. Thus, it is important to recognize the variety of skin reactions, and differences in the treatment paradigm, so that it can help reduce unnecessary interruption of the immunotherapy.
